# Pure tone audiometry and cerebral pathology in healthy older adults

**DOI:** 10.1136/jnnp-2019-321897

**Published:** 2019-11-07

**Authors:** Thomas Parker, David M Cash, Chris Lane, Kirsty Lu, Ian B Malone, Jennifer M Nicholas, Sarah James, Ashvini Keshavan, Heidi Murray-Smith, Andrew Wong, Sarah Buchannan, Sarah Keuss, Carole H Sudre, David Thomas, Sebastian Crutch, Doris-Eva Bamiou, Jason D Warren, Nick C Fox, Marcus Richards, Jonathan M Schott

**Affiliations:** 1 The Dementia Research Centre, Department of Neurodegenerative Disease, UCL Institute of Neurology, London, UK; 2 Department of Medical Statistics, London School of Hygiene and Tropical Medicine, London, UK; 3 MRC Unit for Lifelong Health and Ageing at UCL, UCL, London, UK; 4 KCL School of Biomedical Engineering and Imaging Sciences, London, UK; 5 Neuradiological Academic Unit, UCL, London, Greater London, UK; 6 UCL Ear Institute and UCLH Biomedical Research Centre, National Institute for Health Research, UCL, London, UK

**Keywords:** Alzheimer's disease, amyloid, cognition, image analysis, vascular dementia

## Abstract

**Background:**

Hearing impairment may be a modifiable risk factor for dementia. However, it is unclear how hearing associates with pathologies relevant to dementia in preclinical populations.

**Methods:**

Data from 368 cognitively healthy individuals born during 1 week in 1946 (age range 69.2–71.9 years), who underwent structural MRI, ^18^F-florbetapir positron emission tomography, pure tone audiometry and cognitive testing as part of a neuroscience substudy the MRC National Survey of Health and Development were analysed. The aim of the analysis was to investigate whether pure tone audiometry performance predicted a range of cognitive and imaging outcomes relevant to dementia in older adults.

**Results:**

There was some evidence that poorer pure tone audiometry performance was associated with lower primary auditory cortex thickness, but no evidence that it predicted in vivo β-amyloid deposition, white matter hyperintensity volume, hippocampal volume or Alzheimer’s disease-pattern cortical thickness. A negative association between pure tone audiometry and mini-mental state examination score was observed, but this was no longer evident after excluding a test item assessing repetition of a single phrase.

**Conclusion:**

Pure tone audiometry performance did not predict concurrent β-amyloid deposition, small vessel disease or Alzheimer’s disease-pattern neurodegeneration, and had limited impact on cognitive function, in healthy adults aged approximately 70 years.

## Introduction

Hearing impairment may be a modifiable risk factor for Alzheimer’s disease (AD) and dementia.^1–3^ However, research investigating the relationship between hearing and biomarkers of dementia-associated pathologies in preclinical populations has been limited. Improved understanding of this relationship is important to determine mechanistic relationships, and to evaluate hearing as a screening tool for dementia-associated pathologies. We investigated whether pure tone audiometry performance related to imaging biomarkers of β-amyloid deposition (Aβ), small vessel disease, grey matter macrostructure and cognitive performance in a cohort of healthy older adults all born in the same week of 1946.

## Methods

We included data from 368 cognitively healthy participants drawn from Insight-46, a substudy of the MRC National Survey of Health and Development (NSHD).^4^ All individuals were born during 1 week in 1946.

Positron emission tomography (PET) and MRI data were acquired on the same 3-Tesla PET/MRI scanner.^4^ Aβ-burden was assessed over a 10-min period approximately 50 min after florbetapir injection (370 MBq). A standard uptake value ratio (SUVR) from a grey matter cortical composite using a white matter reference without partial volume correction was calculated. Positive or negative Aβ-status was determined using Gaussian mixture modelling (SUVR cut-point=0.6104).

MRI data included volumetric T1-weighted, T2-weighted and FLAIR images.^4^ Cortical thickness estimation was performed using Freesurfer V.6.0.^5^ A priori regions of interest were: primary auditory cortex (transverse temporal gyrus); planum temporale; and a surface-area weighted AD-signature composed of entorhinal, inferior temporal, middle temporal and fusiform regions.^6^ Hippocampal volume was estimated using STEPS.^7^ Global white matter hyperintensity volume (WMHV—a marker of small vessel disease) was estimated using BaMoS.^8^ Total intracranial volume (TIV) was calculated using Statistical Parametric Mapping 12.

A detailed neuropsychology testing battery was performed^4^ including: the mini-mental state examination (MMSE); digit-symbol substitution; logical memory delayed recall; matrix reasoning; and the 12-item face-name associative memory task.

An otological history regarding hearing aid use, recent ear pain/discharge, tinnitus and previous otological diagnoses was collected. Otoscopy was not performed.

Audiometric thresholds were obtained for each ear at 0.5, 1, 2, and 4 kHz using calibrated Maico-MA-25 audiometers with sound-excluding TDH-49 earphones in audiocups using a British Society of Audiology recommended testing protocol^9^ in a quiet room. A pure-tone average (PTA) in the better hearing ear was calculated using thresholds for 0.5, 1, 2 and 4 kHz.

Wilcoxon rank sum tests were used to assess unadjusted association between binary demographic variables and PTA. Spearman correlations were used to assess unadjusted associations between continuous demographic variables and PTA.

Receiver operating characteristic (ROC) curves and area under the curve (AUC) analyses were performed to investigate whether PTA predicted Aβ-status following adjustment for: age at scan; sex; WMHV; educational attainment^4^; prospective childhood cognition^4^; adult and childhood socioeconomic position.^4^ Additional analyses incorporating *APOE* genotype (binarised into ε4 carrier and non-carriers) were also performed.

As WMHV was non-normally distributed, generalised linear models using the gamma distribution and log link were used to investigate whether PTA predicted WMHV.

Linear regression models with robust standard errors were used to investigate whether PTA predicted grey matter macrostructure (dependent variables=primary auditory cortex, planum temporale AD-signature thickness and hippocampal volume).

Linear regression models with robust standard errors were used to test the hypothesis that PTA predicted cognitive testing performance. For cognitive tests with skewed distributions (MMSE and Matrix Reasoning) bootstrapping with 2000 replications was used to produce bias-corrected and accelerated 95% CIs.

Analyses of WMHV, grey matter macrostructure and cognition were adjusted for age, sex, educational attainment, childhood cognitive ability, socioeconomic position, *APOE* genotype and Aβ-status. Additional adjustment for TIV was made in WMVH and hippocampal volume analyses, while analysis of grey matter macrostructure and cognition were additionally adjusted for WMHV.

Exclusions from 502 participants originally recruited included: failure to complete scan (n=31); PET acquisition failure (n=8); WMHV segmentation error (n=4); MRI movement artefact (n=3); mild cognitive impairment, dementia and/or a major neurological disorder (n=48); history of otological pathology (eg, Meniere’s disease, surgery/trauma resulting in hearing loss, intercurrent infection) (n=16); equipment unavailable (n=19); missing *APOE* genotype (n=2); missing socioeconomic position (n=3).

## Results

As all individuals were born in the same week, the age range at scanning was narrow (69.2–71.9 years). Median PTA across the dataset was 22.5 dB HL (decibels in hearing level) (IQR=12.5). Unadjusted associations between PTA and a range of variables are displayed in table 1. Of note, both tinnitus and hearing aid use were strongly associated with higher PTA (table 1).

**Table 1:** Summary statistics for auditory testing/imaging biomarkers and unadjusted relationships between demographic and performance on pure tone audiometry AND associations between pure tone audiometry performance and neuropsychological testing in cognitively normal older adults

Binary demographics	Unadjusted association with PTA		
	Median (IQR) PTA		P value
Sex	Males: 23.5 (12.5), n=187 Females: 21.25 (12.5), n=181		0.19*
Educational attainment	Non-advanced: 23.75 (15), n=169 Advanced: 21.25 (10), n=199		0.09*
Childhood socioeconomic position	Non-manual: 22.55 (11.25), n=218 Manual: 21.25 (15), n=150		0.95*
Adulthood socioeconomic position	Non-manual: 21.25 (11.25), n=313 Manual: 23.75 (16.25), n=55		0.17*
Tinnitus	**No tinnitus: 21.25 (12.5), n=** **288** **Tinnitus: 26.25 (13.75), n=** **80**		0.0004*
Hearing aid use	**Non-user: 21.25 (10), n=** **313** **User: 38.75 (16.25), n=** **55**		<0.0001*
*APOE* genotype	Non-ε4 carrier: 22.5 (12.5), n=258 ε4 carrier: 21.25 (13.25), n=110		0.089*
Aβ positivity, n (%)	Aβ-negative: 21.88 (12.5), n=306 Aβ-positive: 23.75 (12.5), n=62		0.25*
Continuous demographics	**Summary statistic**	**Unadjusted association with PTA**	
Age, years, median (IQR)	70.6 (1.1), n=368	Rho=0.07, p=0.2†	
Childhood cognition, z-score, median (IQR)	0.48 (0.84), n=368	Rho=−0.02, p=0.72†	
Cognitive tests	**Summary statistic**	**Association with PTA**	
		**Univariate**	**Multivariate β-coefficient‡ (95% CI**)
Logical memory delayed recall (Wechsler Memory Scale-Revised—out of 25), mean (SD)	11.6 (3.6), n=368	r=−0.065, p = 0.21§	−0.009 (−0.044 to 0.026)¶
Digit-symbol substitution (Wechsler Adult Intelligence Scale-Revised—out of 93), mean (SD)	48.8 (10.0), n=368	r=−0.047, p = 0.36§	−0.005 (−0.098 to 0.087)¶
12-item Face-Name (out of 96), mean (SD)	65.6 (18.2), n=364	r=0.099, p = 0.85§	−0.01 (−0.20 to 0.18)¶
Matrix Reasoning (Wechsler Abbreviated Scale of Intelligence—out of 32), median (IQR)	25 (4), n=368	Rho=−0.11, p=0.04§	−0.037 (−0.084 to 0.007)**
MMSE (out of 30), median (IQR)	30 (1), n=368	Rho=−0.13, p=0.013§	−**0.009** (**−0.019 to** **−0.001**)**
MMSE without ‘No ifs, ands or buts’ (out of 29), median (IQR)	29 (1), n=368	Rho=−0.07, p=0.21§	−0.005 (−0.015 to 0.003)**
“No ifs, ands or buts” repetition (out of 1), proportion who answered Item correctly (%)	306/368, (83.2%)	p=0.0017^a^	−**0.031** (**−0.056 to** **−** **0.006**)††

Analyses that attained statistical significance at the 5% level are highlighted in bold.

*Wilcoxon rank sum test.

†Spearman correlation.

‡Coefficient represents increase in cognitive score per dB HL increase in PTA (covariates were: age at scan; sex; APOEε4 genotype; WMHV; educational attainment; childhood cognitive ability; socioeconomic position; and Aβ status).

§Pearson’s correlation.

¶Linear regression models with robust SE.

**Bootstrapping.

††Logistic regression.

Aβ, β-amyloid; AD, Alzheimer’s disease; MMSE, mini-mental-state-examination; PET, positron emission tomography; PTA, pure tone average in best hearing ear; r, Pearson’s r; Rho, Spearman’s Rho; WMHV, white matter hyperintensity volume.

A total of 62 participants were Aβ-positive (16.8%). Unadjusted analyses revealed no evidence of an association between Aβ and PTA (table 1). Using a base model combining age, sex, WMHV, educational attainment, childhood cognitive ability and socioeconomic position, ROC analysis provided an AUC for Aβ-positivity of 0.55 (95% CI 0.48 to 0.63); subsequent inclusion of PTA (AUC=0.56, 95% CI 0.49 to 0.64) did not improve the predictive ability. Incorporating *APOE* genotype into the base model provided an AUC for Aβ-positivity of 0.70 (95% CI 0.62 to 0.78). Again, inclusion of PTA (AUC=0.72; 95% CI 0.64 to 0.79) did not improve this.

There was no evidence that PTA predicted WMHV in fully adjusted models (exponentiated coefficient=0.96; 95% CI 0.98 to 1.01; p=0.39, which corresponds to the proportional change in WMHV per dB HL increase in PTA).

**Figure 1:**
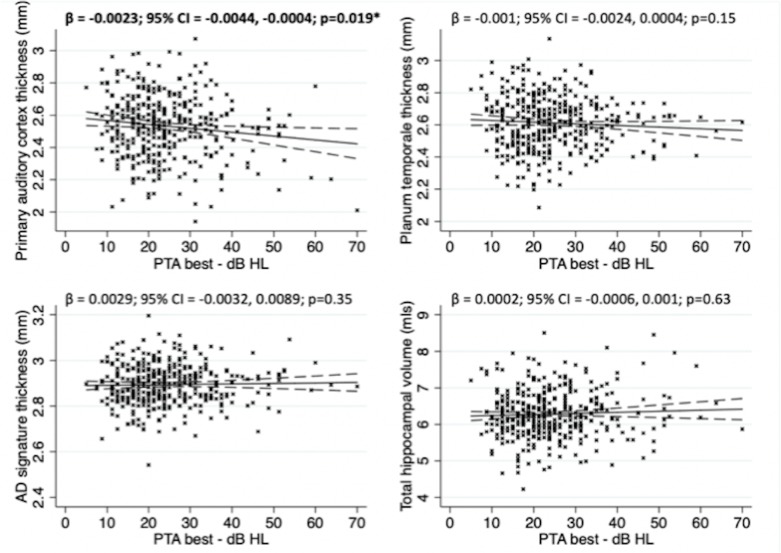
Association between pure tone audiometry performance and grey matter structural metrics in cognitively normal older adults following multivariate linear regression with robust SE. Covariates included: age at scan; sex,; *APOE*ε4 genotype; WMHV; educational attainment; childhood cognitive ability; socioeconomic position’Aβ status; and TIV (for hippocampal volume analysis only). Solid line represents line of best of fit. Dashed lines represent 95% CIs. The crosses represent raw unadjusted data points for each individual included in the analysis. AD, Alzheimer’s disease; PTA, pure tone average in best hearing ear; TIV, total intracranial volume; WMHV, white matter hyperintensity volume.

There was evidence of a negative association between primary auditory cortex thickness and PTA (figure 1). No associations were observed between PTA and planum temporale thickness, AD-signature thickness or hippocampal volume (figure 1).

There was no evidence of a relationship between PTA and a range of cognitive scores following covariate adjustment, except for a negative association between PTA and MMSE. However, this was no longer evident when excluding the phrase repetition item ‘no ifs, ands, or buts’ (table 1).

A post-hoc analysis using high frequency thresholds only (4 kHz rather than a PTA) was performed and produced very similar results (data not shown).

## Discussion

We found no evidence of an association between PTA and Aβ-deposition in cognitively healthy individuals aged 69.2–71.9 years. To our knowledge, this is the first study to investigate the relationship between hearing and PET-derived measures of Aβ, although a null relationship between cerebrospinal fluid biomarkers of Aβ-deposition and auditory function has been reported in healthy older adults with a family history of AD.^10^ There was also no evidence of a relationship between PTA and WMHV, which to our knowledge has not been investigated previously. Taken together these results suggest impaired hearing is not a reliable marker of preclinical Aβ-deposition or small vessel disease, at least early in the eighth decade. This is supported by a recent systematic review and meta-analysis, which found evidence that hearing impairment increased the risk of cognitive impairment and all-cause dementia, but did not increase the risk for AD or vascular dementia specifically.^11^

Beyond Aβ and small vessel disease, another potential pathophysiological mechanism linking hearing and dementia is neurodegeneration. Hearing loss has been previously shown to be associated with grey matter atrophy including regions typically affected in AD.^10 12^ However, in our analyses, neither hippocampal volume nor AD-cortical signature thickness were associated with PTA, suggesting that hearing impairment is unlikely to be related to AD-pattern neurodegeneration in healthy older adults. In contrast we found evidence for an independent association between PTA and primary auditory cortex thickness, which has been shown previously,^13^ but not with consideration of Aβ and WMHV. Although speculative, this would be consistent either with a non-AD and non-vascular related degeneration of this region contributing to hearing impairment, or with peripheral deafferentation of the cochlear nerve due to presbycusis leading to atrophy in primary auditory areas, although the cross-sectional nature of the data limits ability to infer a causal direction. Furthermore, the association observed was cross-sectional and relatively weak statistically following adjustment for covariates necessitating cautious interpretation.

In terms of cognition, the only significant finding was an association between PTA and MMSE score, which was no longer evident when a test item assessing repetition of a single phrase (‘no ifs, ands or buts’) was excluded, suggesting that although hearing ability had limited impact on overall cognitive function, hearing loss may impact acoustically demanding tests and is an important consideration while administering and interpreting such tests.

Limitations include: its cross-sectional nature; missing data; lack of a more detailed otological examination; and inability to precisely define causes of hearing loss. Future work of importance will include inclusion of longitudinal auditory, cognitive and neuroimaging data, and long-term follow-up. It is possible that auditory dysfunction *does* precede the development of cognitive impairment in AD, but that this occurs sometime after Aβ-deposition; individuals in this study are aged 69.2–71.9 years and may be many years away from developing cognitive impairment, and the downstream pathological changes associated with hearing impairment. Neuroimaging techniques that assess other pathological process relevant to dementia such as tau-PET,^14^ may provide further insights. Including a broader range of auditory testing, specifically including tests that focus on central auditory processing,^15^ which is known to be impaired in established AD will also be of considerable interest. The majority of participants investigated had normal hearing thresholds, which may limit the power to detect relationships between hearing function and outcomes relevant to dementia. However, the statistically significant relationships observed between tinnitus, hearing aid use and primary auditory cortex thickness would support the notion that this study is sufficiently powered to detect meaningful relationships between hearing function and outcomes of interest.

In summary, we demonstrate that pure tone audiometry performance did not predict concurrent Aβ-deposition, small vessel disease or AD-pattern neurodegeneration, and had limited impact on cognitive function, in healthy adults aged approximately 70 years.

## References

[1] Livingston G, Sommerlad A, Orgeta V, et al Dementia prevention, intervention, and care. The Lancet 2017;390:2673–734. 10.1016/S0140-6736(17)31363-6 28735855

[2] Deal JA, Betz J, Yaffe K, et al Hearing impairment and incident dementia and cognitive decline in older adults: the health ABC study. J Gerontol A Biol Sci Med Sci 2017;72:703–9. 10.1093/gerona/glw069 27071780 PMC5964742

[3] Lin FR, Albert M Hearing loss and dementia – who is listening? Aging Ment Health 2014;18:671–3. 10.1080/13607863.2014.915924 24875093 PMC4075051

[4] Lane CA, Parker TD, Cash DM, et al Study protocol: insight 46 – a neuroscience sub-study of the MRC national survey of health and development. BMC Neurol 2017;17 10.1186/s12883-017-0846-x PMC5395844 28420323

[5] Fischl B, Dale AM Measuring the thickness of the human cerebral cortex from magnetic resonance images. Proc Natl Acad Sci U S A 2000;97:11050–5. 10.1073/pnas.200033797 10984517 PMC27146

[6] Jack CR, Wiste HJ, Weigand SD, et al Different definitions of neurodegeneration produce similar amyloid/neurodegeneration biomarker group findings. Brain 2015;138:3747–59. 10.1093/brain/awv283 26428666 PMC4655341

[7] Jorge Cardoso M, Leung K, Modat M, et al Steps: similarity and truth estimation for propagated segmentations and its application to hippocampal segmentation and brain parcelation. Med Image Anal 2013;17:671–84. 10.1016/j.media.2013.02.006 23510558

[8] Sudre CH, Cardoso MJ, Bouvy WH, et al Bayesian model selection for pathological neuroimaging data applied to white matter lesion segmentation. IEEE Trans Med Imaging 2015;34:2079–102. 10.1109/TMI.2015.2419072 25850086

[9] British Society of Audiology Recommended procedure bone-conduction threshold audiometry with and without masking 2011.

[10] Tuwaig M, Savard M, Jutras B, et al Deficit in Central Auditory Processing as a Biomarker of Pre-Clinical Alzheimer’s Disease. JAD 2017;60:1589–600. 10.3233/JAD-170545 28984583 PMC5757649

[11] Loughrey DG, Kelly ME, Kelley GA, et al Association of age-related hearing loss with cognitive function, cognitive impairment, and dementia: a systematic review and meta-analysis. JAMA Otolaryngol Head Neck Surg 2018;144 10.1001/jamaoto.2017.2513 PMC5824986 29222544

[12] Lin FR, Ferrucci L, An Y, et al Association of hearing impairment with brain volume changes in older adults. Neuroimage 2014;90:84–92. 10.1016/j.neuroimage.2013.12.059 24412398 PMC3951583

[13] Peelle JE, Troiani V, Grossman M, et al Hearing loss in older adults affects neural systems supporting speech comprehension. J Neurosci 2011;31:12638–43. 10.1523/JNEUROSCI.2559-11.2011 21880924 PMC3175595

[14] Johnson KA et al Tau positron emission tomographic imaging in aging and early Alzheimer disease. Ann. Neurol 2016. 10.1002/ana.24546 PMC4738026 26505746

[15] Gates GA, Anderson ML, McCurry SM, et al Central auditory dysfunction as a harbinger of Alzheimer dementia. Arch Otolaryngol Head Neck Surg 2011;137:390–5. 10.1001/archoto.2011.28 21502479 PMC3170925

